# Leveraging immune and clinicopathological profiles with machine learning to predict axillary lymph node metastasis in breast cancer patients

**DOI:** 10.1186/s13058-026-02294-9

**Published:** 2026-05-08

**Authors:** Alba Fischer-Carles, Alessio Fiorin, Carlos López Pablo, Laia Reverté, Esther Sauras-Colón, Noèlia Gallardo-Borràs, Marylène Lejeune, Mikel Relloso Ortiz de Uriarte, Tábata Sánchez-Alcántara, Maria Ballester-Navarro, Ramon Bosch Príncep, Laia Adalid-Llansa, Elena Goyda, Daniel Mata Cano, Jérôme Noailly, Gemma Piella

**Affiliations:** 1 Oncological Pathology and Bioinformatics Research Group, Institut de Recerca Biomèdica Catalunya Sud (IRB CatSud), Tortosa, Spain; 2 Department of Pathology, Hospital Universitari de Tortosa Verge de la Cinta, Institut Català de la Salut, Tortosa, Spain; 3 Department of Computer Engineering and Mathematics, Universitat Rovira i Virgili, Tarragona, Spain; 4 Department of Engineering, BCN MedTech, Universitat Pompeu Fabra, Barcelona, Spain; 5 Department of Pathology, Hospital Universitari Joan XXIII, Institut Català de la Salut, Tarragona, Spain

**Keywords:** Breast cancer, Machine learning, Immune response, Clinicopathological data, Axillary lymph node metastasis

## Abstract

**Background:**

Breast cancer is the leading cause of cancer-related death in women, with mortality increasing when tumor cells spread to nearby lymph nodes, particularly the axillary lymph nodes (ALNs). Although several studies predict patients with ALN metastasis at diagnosis (pdALN^+^), few examine the prognostic value of immune elements within ALNs. Given the impact of immune response on breast cancer, this study develops a machine learning model to identify the clinicopathological and immune features of the primary tumor and non-metastatic ALNs (ALN^−^) most frequently associated with pdALN^+^.

**Methods:**

Two datasets of luminal breast cancer patients diagnosed between 1995 and 2008 were used: Dataset 1 involved 83 women (42 pdALN^−^ and 41 pdALN^+^), and Dataset 2 comprised 344 women (204 pdALN^−^ and 140 pdALN^+^). Three machine learning models were developed using the Random Forest algorithm: Model 1 included clinicopathological data from Dataset 1; Model 2 used clinicopathological and immune response data from Dataset 1; and Model 3 used clinicopathological data from Dataset 2. All models followed the same machine learning pipeline, including data pre-processing, feature selection using recursive feature elimination with cross-validation, algorithm optimization using random search cross-validation, and results interpretability using Shapley additive explanations values. After selecting the best-performing model, Model 4 was developed using its dataset and features. The optimal feature set was determined at the point where adding more features led to a decline in model performance metrics.

**Results:**

Model 2 outperformed Models 1 and 3, despite the larger cohort on which Model 3 was developed. This highlights the crucial role of the immune response in breast cancer progression. Model 4 achieved a median ROC AUC of 0.84, a median accuracy of 0.76, and a median recall of 0.75. Remarkably, nine of the ten predictive features were immune populations. The intratumoral follicular dendritic cell marker CD21^+^ was the most predictive feature, even surpassing tumor diameter, a well-established prognostic factor in breast cancer. Thus, it might stand as a novel biomarker candidate.

**Conclusions:**

This study not only identifies promising biomarker candidates but also highlights the importance of including mechanistic features, such as mediating inflammation, in breast cancer patient stratification.

**Supplementary Information:**

The online version contains supplementary material available at 10.1186/s13058-026-02294-9.

## Background

Breast cancer (BC) is the most common malignant tumor among women, and one of their leading causes of mortality. In 2022, BC accounted for 23.8% of all female cancers worldwide and 15.4% of cancer-related deaths [1]. BC survival rate declines when tumor cells spread beyond the primary site, producing regional or distant metastases [2]. Regional metastasis usually occurs in the axillary lymph node (ALN), and is a key factor associated with worse prognosis in BC patients [3]. The presence of metastasis in ALN (ALN^+^) increases the risk of developing distant metastasis [4], which is the leading cause of death in cancer patients [5]. In addition to ALN status and other well-established prognostic factors such as tumor size and molecular subtype, emerging evidence draws attention to the important role of the intratumoral immune response in patient evolution, response to treatments, and survival [6–8]. Although ALNs are neuralgic centers of the immune response of the body and primary sites of BC regional metastasis, few studies have investigated the prognostic value of immune elements in these nodes [9, 10]. Previous studies addressed this knowledge gap by evaluating the expression of immune markers in both the primary tumor and non-metastatic ALN (ALN^−^) [11–13]. They revealed that the presence of certain immune cells in the ALN^−^ could be related to specific molecular profiles, patient outcomes, and the presence of neighboring ALN^+^. In these studies, the immune profiling of ALNs relied on conventional statistical methods, such as Cox regression, logistic regression, and Kaplan–Meier survival analyses [14].

In recent years, artificial intelligence (AI), particularly machine learning (ML), has prompted remarkable advances in cancer research [15, 16], enabling early-stage cancer detection, facilitating the development of personalized treatments, and predicting disease progression or treatment response, among other achievements [17]. ML encompasses various techniques capable of identifying patterns and continuously learning from making predictions and decisions of their own [18]. Deep learning, a further subset of ML, involves neural networks with many layers and typically requires extensive datasets in order to perform well [19]. However, acquiring a comprehensive medical dataset is challenging due to the need to ensure data privacy, regulatory constraints, the high costs associated with accessing clinical data, and the difficulty of dealing with non-structured clinical records [20, 21]. Some techniques, such as Random Forest (RF), can robustly handle smaller datasets, and are the preferred ML solutions when data are limited [22].

Overall, AI is showing promising results in BC patient stratification, and current research increasingly uses AI models to predict ALN status (presence or absence of metastasis in the ALNs) using clinicopathological features [23, 24]. However, the immune response is rarely considered, and no previous studies have combined ML with the clinicopathological features and immune information of the primary tumor and ALN^−^. The immune response in the primary tumor and ALNs of BC patients is not routinely evaluated in clinical practice, limiting the availability of pre-existing data, creating a barrier to the exploitation of some ML techniques and of AI that would enable patient outcomes to be predicted [25]. Despite these challenges, the ability of tumor cells to alter the immune response in the primary tumor and the ALNs makes it genuinely necessary to discern the immune patterns between patients with and without ALN metastasis at diagnosis (pdALN^+^ vs. pdALN^−^). Accordingly, the present study aims to identify the clinicopathological features and immune populations most strongly associated with pdALN^+^ by exploring the specific types of ML models designed to handle small quantities of data for patient stratification in BC.

## Methods

### Study design

This study included two datasets: Dataset 1 involved 83 women diagnosed between 1995 and 2008 with invasive breast carcinoma of no special type, and Dataset 2 comprised 344 women (83 from Dataset 1) diagnosed between 1995 and 2019 with invasive breast carcinoma of no special type. A ten-year follow-up period was established for both datasets. The inclusion criteria were: 1) no distant metastasis at diagnosis, 2) no missing data, and 3) the case was hormone receptor-positive (luminal BC subtype). In Dataset 1, 72 and 11 of the 83 BC patients were, respectively of the Luminal A (86.7%) and Luminal B (13.3%) subtypes. In Dataset 2, 204 and 140 of the 344 BC patients were of the Luminal A (59.3%) and Luminal B (40.7%) subtypes, respectively. The subtypes were assigned in accordance with the World Health Organization guidelines for classifying BC in force at the time of the study [26]. At diagnosis, patients were categorized into two groups depending on their ALN status: 42 pdALN^−^ (50.6%) and 41 pdALN^+^ (49.4%) in Dataset 1, and 204 pdALN^−^ (59.3%) and 140 pdALN^+^ (40.7%) in Dataset 2. Furthermore, 10.8% of patients from Dataset 1 and 3.8% of patients from Dataset 2 received neoadjuvant therapy. ALN status was determined based on pathological assessment of surgically excised lymph nodes. Patients were classified as pdALN^+^ or pdALN^−^ according to the presence or absence of metastasis in the examined ALN. In patients receiving neoadjuvant therapy, nodal status reflected post-treatment pathology (ypN), whereas in patients undergoing upfront surgery it reflected standard pathological staging at primary resection (pN). Clinically node-negative patients typically underwent sentinel lymph node biopsy, while patients with nodal metastases underwent completion axillary lymph node dissection according to standard clinical practice during the study period (1997–2008).

### Dataset composition

Dataset 1 consisted of clinicopathological and immune data, whereas Dataset 2 comprised exclusively clinicopathological data. Immune data were obtained from the biopsies of the primary tumor and the ALN^−^ from pdALN^+^ and pdALN^−^ from the Tumor Banks (Biobank IISPV) of the Hospital Universitari de Tortosa Verge de la Cinta (HUTVC) and the Hospital Universitari Joan XXIII (HJ23), Tarragona, Spain. The immune markers were studied via tissue microarrays, immunohistochemical (IHC) staining, digitization of stained slides, and analysis of digitized images to quantify the IHC markers using methods established in previous studies [11, 12]. Eleven immune populations were evaluated: CD4^+^ helper T lymphocytes, CD8^+^ cytotoxic T lymphocytes, CD57^+^ natural killer cells, FOXP3^+^ regulatory T cells, CD21^+^ follicular dendritic cells (DCs), CD68^+^ macrophages, CD1a^+^ Langerhans DCs, CD123^+^ plasmacytoid DCs, S100^+^ interdigitant DCs, LAMP3^+^ DCs, and CD83^+^ mature DCs. The S100^+^ interdigitant DCs from the primary tumor were excluded because many of the data were missing, and its relevance had not been demonstrated in earlier studies [11]. The immune population was quantified by determining the percentage of the positively stained area occupied by immune cells relative to the overall tissue area using digital image analytical procedures as described in other studies [27, 28]. Although the cohorts of this study predate the standardized reporting criteria [7], representative intratumoral regions of the primary tumor containing sufficient tumor cellularity were consistently selected for immune cell assessment. Regions with central sclerosis or poor cellular representation were avoided, and immune populations were quantified relative to the area within the tumor perimeter. Peritumoral regions were not included, as these have been analyzed separately in previous studies [27].

Clinicopathological data from both datasets were collected by reviewing pathology reports and clinical details in patients’ electronic medical records. These data included age at diagnosis (years), tumor diameter (mm), histological grade (I, II, or III), perineural invasion (yes or no), estrogen receptor expression (positive or negative), progesterone receptor expression (positive or negative), human epidermal growth factor receptor 2 (HER2) amplification (amplified or not-amplified), proliferation index (Ki67) (low, medium, or high), and ALN status (positive or negative). As in our earlier research [11], lymphovascular invasion was excluded from the study because it tends to be overestimated by pathologists.

The present study received approval from the Research Committee of the HUTVC and the Ethics Committee of HJ23 in Tarragona (reference number 22p/2011) and the Ethics Committee of the IISPV (reference numbers 128/2022 & 218/2022). Written informed consent was obtained from all patients for their involvement in the study and for using their biopsy specimens and clinical data. The study adhered to the Strengthening the Reporting of Observational Studies in Epidemiology (STROBE) guidelines and to the standards of the Declaration of Helsinki.

### Methodology

**Fig. 1:**
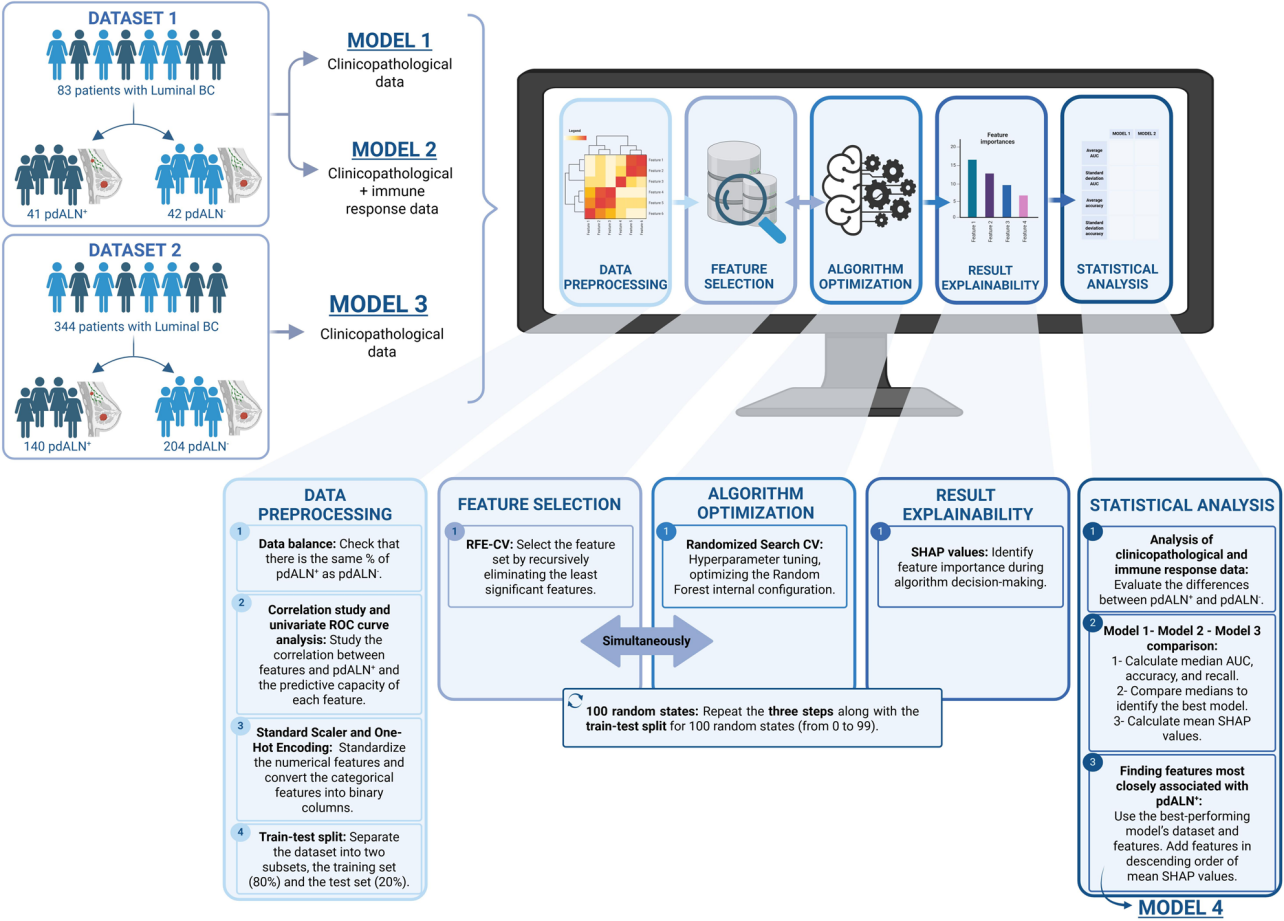
Outline of the study design. Description of patient cohorts and the steps in the computational procedure (data preprocessing, feature selection, algorithm optimization, and explainability of the results) and statistical analysis. Created in BioRender.com

Four supervised ML models were developed. Model 1 integrated exclusively clinicopathological data from Dataset 1, while Model 2 combined both clinicopathological and immune data from Dataset 1. Therefore, Models 1 and 2 used the same cohort of patients but different input data. In contrast, Model 3 used clinicopathological data from Dataset 2. To ensure the fair comparison of the results, the same computational procedure and statistical analysis were used for all three models (Fig. 1). Finally, after selecting the best-performing model, Model 4 was developed using the same dataset and features as the best model.

All the scripts were written in the Python programming language (version 3.11.4). NumPy 1.24.3 [29] was used for numerical computations, while data were manipulated and analyzed using Pandas 1.5.3 [30]. Visualization tasks were managed with Matplotlib 3.7.1 [31]. Models were interpreted with SHAP 0.42.1 [32], and ML tasks were performed with Scikit-learn 1.2.2 [33]. Imbalanced-learn 0.10.1 [34] was chosen to address class imbalance issues. Advanced mathematical functions were supported by SciPy 1.10.1 [35]. Statistical analysis tests were carried out with IBM SPSS Statistics 23.0 (IBM, Armonk, NY, USA).

### Computational procedure

The computational procedure involved preprocessing data and developing the ML models, which in turn included feature selection, algorithm optimization, and result explainability.

#### Data preprocessing

Initially, the study involved 181 individuals from Dataset 1 and 465 individuals from Dataset 2 diagnosed with various BC subtypes, including luminal (Luminal A and Luminal B), HER2-positive, and triple-negative BC. Since luminal BC is the most commonly diagnosed and homogeneous BC subtype, the study focused on this subtype to enhance uniformity and improve model performance [36]. Consequently, Datasets 1 and 2 were reduced from 181 to 106 and from 465 to 348 individuals, respectively, by selecting only the hormone receptor-positive patients. Individuals with incomplete information were excluded since implementing missing data techniques, such as k-Nearest Neighbor Imputer [37] or Iterative Imputer [38], introduced significant errors and worsened the quality of the data. This process gave a final cohort of 83 individuals in Dataset 1 and 344 individuals in Dataset 2, as detailed in the *Study design* section. Furthermore, Spearman correlation was employed to explore correlations between features and pdALN^+^, and a univariate Receiver Operating Characteristic (ROC) curve analysis was done to evaluate the predictive capacity of each feature individually.

Data standardization is crucial to the performance of ML models, specifically when dealing with large-scale features. Thus, a standard scaler method was employed to transform the distribution of each feature to give a mean of 0 and a standard deviation of 1 [39]. In addition, categorical features were converted into binary vectors through one-hot encoding to facilitate their integration [40].

Once the numerical data had been standardized, the dataset was split into training and test subsets. The training set contained 80% of the data (66 patients from Dataset 1 and 275 from Dataset 2), and the test set comprised the remaining 20% (17 patients from Dataset 1 and 69 from Dataset 2). The division was stratified, maintaining the proportion of the study feature (the pdALN^+^) in both subsets. This dataset split aimed to enhance the reliability of the model, as training and testing were performed on two independent subsets to prevent overfitting and to evaluate generalizability with the test subset [41].

#### Machine learning model development

Selecting a suitable ML algorithm improves prediction metrics. In this study, the RF classifier was used to evaluate the features associated with pdALN^+^ [42]. RF combines multiple decision tree classifiers, each trained on a random subset of the dataset, to enhance predictive accuracy. Three fundamental ML steps were implemented to improve the performance of the RF classifier: A) feature selection, B) algorithm optimization, and C) result explainability.

A) **Feature selection**: Choosing the proper set of features improves model performance. For this reason, we first evaluated several feature selection methods, including Sequential Feature Selector and Recursive Feature Elimination with Cross-Validation (RFE-CV), both employing Area Under the Curve (AUC) from the ROC curve and accuracy scoring, as well as Variance Inflation Factor, and L1-Regularized Logistic Regression. AUC scoring with RFE-CV performed slightly better than the other methods (Supplementary material Table S1). The RFE-CV combines the advantages of RFE and CV, and systematically determines the importance of features by recursively removing the least significant factor in the output prediction, employing CV and a scoring method to assess the model’s performance at each iteration [43]. In this case, AUC scoring and stratified K-Fold Cross-Validation (K-Fold CV) were used, particularly 3-Fold CV.

B) **Algorithm optimization**: In this second step, the hyperparameters, which are the ML algorithm’s configuration parameters that affect its operation and performance, were optimized to improve the efficacy of RF when predicting pdALN^+^ [42]. Several hyperparameters were carefully adjusted, including: the number of trees; the number of features that allow the best split; the maximum depth of a tree; the minimum number of samples required to split an internal node and be at a leaf node; whether bootstrap samples were used when building trees. These hyperparameters were tuned using the Randomized Search CV method [44], which is less time-consuming, allows broader exploration, and is more adaptable than other methods, such as Grid Search CV [45, 46].

Feature selection and algorithm optimization were performed simultaneously. This integrated approach optimizes the model while identifying the most relevant features using only the training set (80% of the data).

C) **Result explainability**: From the perspective of ML algorithms, explainability involves understanding the weight attributed to each feature in the final model when discerning whether a patient has ALN^+^ at diagnosis. SHapley Additive exPlanations (SHAP) values were used to achieve this. Their implementation allows the impact of individual features on the model predictions to be interpreted, revealing the features that most strongly contribute to the decision-making [47].

The RFE-CV, the RF, and the train-test split require a random state to be introduced to ensure that results are replicable. The selected random state determines the division of the dataset and influences the initial choice of features and patients for exploration via RFE-CV and RF. If the same random state is used, the results will be the same for identical inputs. However, choosing a random state in small datasets can introduce variability in the outcome. To minimize the impact of the random state on result variability, given the small size of the dataset, the steps of feature selection, algorithm optimization, and result explainability, along with splitting the training and test subsets, were repeated for 100 different random states, ranging from 0 to 99. This approach aims to evaluate the overall trend in the results, allowing for more robust interpretations to be made.

### Statistical analysis

The differences between the concentrations of the immune populations evaluated in the primary tumor and the ALN^−^ were assessed by Student’s independent samples t-test or the Mann–Whitney U-test, depending on whether data were normally distributed. The same tests were used to evaluate differences in quantitative clinicopathological features. To examine differences between classes of categorical clinicopathological features, the chi-square or Fisher’s exact test was employed, depending on the sample size.

The features with the highest AUC from the univariate ROC curve analysis when predicting pdALN^+^ and those with the highest Spearman correlation coefficient with pdALN^+^ were selected, using the top 10 percent, determined from the magnitude of the absolute values of the correlation coefficients, as a cutoff. In the univariate ROC curve analysis, the top 10 percent was selected by transforming AUC values between 0.5 and 1. As AUC values less than 0.5 might indicate reverse predictions, an additional test was conducted to confirm that the model would not predict pdALN^+^ but pdALN^−^, the features also being possible candidates for inclusion in the top 10 percent. For instance, an AUC value of 0.35 when predicting ALN^+^, would become 0.65 for predicting ALN^−^. This 10 percent cutoff was used solely for exploratory purposes, to facilitate visualization and structured discussion of intermediate results, and it was applied consistently throughout the manuscript.

To assess and compare the effectiveness of Models 1, 2 and 3, the AUC, the recall of positive class (sensitivity), and the accuracy of the three models were evaluated based on the results from the 100 repetitions. Since neither the AUC, accuracy, nor recall values were normally distributed, the medians of these 100 results, with their respective interquartile ranges, were calculated for the three models and compared using Friedman’s test [48]. Conover’s test was then performed to identify significant differences between specific pairs (*p*-value < 0.05) [49]. Once the best model had been selected, a final analysis was performed to identify the features most strongly associated with pdALN^+^. For each of these 100 repetitions, we obtained the SHAP value attributed to each feature in the decision-making process. The mean of the 100 SHAP values was then calculated for each feature to determine the overall contribution of the feature to the model predictions. The top 10 percent of these mean values was used as a cutoff to highlight the features with the strongest influence on the model’s decision-making process, thereby facilitating the interpretation and discussion of intermediate results. The dataset and features used in the best-performing model were then used to develop a new model (termed Model 4). Unlike the previous models, features were recursively introduced in descending magnitude of the mean SHAP values from the 100 repetitions for the best-performing model. Each time a new feature was added, it was used with all the previously included features. The entire process, including feature selection, algorithm optimization, result explainability, and train-test split, was then repeated for the 100 different random states. Finally, recall, AUC, and accuracy were calculated for each set of features. The cutoff to identify the features most strongly associated with ALN^+^ presence at diagnosis was declared as the point at which the addition of a new feature reduced the median or mean values of recall, AUC, and accuracy. Once the optimal number of features had been identified, Model 4 was compared with the best-performing model using the Wilcoxon signed-rank test.

## Results

### Data exploration and univariate analysis

First, the balance of the datasets was checked. Dataset 1 was roughly balanced with near-equal numbers of pdALN^+^ and pdALN^−^ cases (Table 1 and Table 2), while Dataset 2 was slightly imbalanced (Table 3).

**Table 1:** Demographic and clinicopathological features of pdALN^-^ and pdALN^+^ from Dataset 1

Variables	pdALN^-^ (*n* = 42)	pdALN^+^ (*n* = 41)	*p*-value
Age (years)	59.55 (10.92)	61.27 (12.02)	0.497^†^
Tumor diameter (mm)	15.00 (9.00)	24.00 (15.00)	**< 0.001** ^**‡**^
Histological grade			
I	16 (38.10%)	3 (7.32%)	0.002*
II	19 (45.24%)	23 (56.10%)	
III	7 (16.66%)	15 (36.58%)	
Perineural invasion			
Positive	7 (16.67%)	18 (43.90%)	0.007*
Negative	35 (83.33%)	23 (56.10%)	
ER expression			
Positive	39 (92.86%)	39 (95.12%)	1.000*
Negative	3 (7.14%)	2 (4.88%)	
PR expression			
Positive	34 (80.95%)	29 (70.73%)	0.276*
Negative	8 (19.05%)	12 (29.27%)	
HER2 expression			
Amplified	6 (14.29%)	5 (12.20%)	0.779*
Non-amplified	36 (85.71%)	36 (87.80%)	
Ki67 degree			
Low	16 (38.09%)	14 (34.15%)	0.714*
Medium	17 (40.48%)	15 (36.58%)	
High	9 (21.43%)	12 (29.27%)	
Molecular subtype			
Luminal A	36 (85.71%)	36 (87.80%)	0.779*
Luminal B	6 (14.29%)	5 (12.20%)	

pdALN^-^ = Patients without Axillary Lymph Node metastasis at diagnosis. pdALN^+^ = Patients with Axillary Lymph Node metastasis at diagnosis. ER = Estrogen Receptor. PR = Progesterone Receptor. HER2 = Human Epidermal growth factor Receptor 2. Ki67 = Proliferation index.

Data from quantitative features are presented as the mean (standard deviation) or median (interquartile range) and analyzed using Student’s t-test^†^ or the Mann–Whitney U-test^‡^ for normally and non-normally distributed data, respectively. Data from qualitative features are presented as absolute frequencies (percentages) and analyzed using the chi-square test or, if any expected frequency was less than 5, Fisher’s exact test*. A *p*-value < 0.05, in bold, indicates statistically significant differences between pdALN^-^ and pdALN^+^.

**Table 2:** Immune population concentrations in the primary tumor and ALN^-^ of pdALN^-^ and pdALN^+^ from Dataset 1

Immune markers	pdALN^-^ (*n* = 42)	pdALN^+^ (*n* = 41)	*p*-value
Primary tumor			
CD4	0.867 (1.691)	0.789 (1.352)	0.518^‡^
CD8	1.090 (1.393)	1.237 (1.897)	0.743^‡^
CD57	0.106 (0.223)	0.171 (0.792)	0.148^‡^
FOXP3	0.108 (0.155)	0.069 (0.144)	0.293^‡^
CD21	0.000 (0.002)	0.016 (0.048)	**< 0.001** ^**‡**^
CD68	2.589 (2.858)	2.736 (1.875)	0.591^‡^
CD1a	0.104 (0.264)	0.113 (0.215)	0.956^‡^
CD123	0.000 (0.004)	0.000 (0.024)	0.432^‡^
LAMP3	0.019 (0.060)	0.059 (0.199)	**0.041** ^**‡**^
CD83	0.105 (0.144)	0.113 (0.219)	0.530^‡^
ALN^**-**^			
CD4	59.404 (12.378)	54.56 (12.42)	0.079^†^
CD8	12.164 (9.324)	15.271 (8.716)	**0.028** ^**‡**^
CD57	0.191 (0.171)	0.179 (0.259)	0.291^‡^
FOXP3	2.053 (1.232)	2.151 (1.846)	0.649^‡^
CD21	0.702 (1.669)	0.701 (1.334)	0.566^‡^
CD68	8.752 (3.637)	10.023 (3.693)	0.118^†^
CD1a	1.620 (3.566)	1.731 (4.039)	0.392^‡^
CD123	1.786 (2.340)	1.197 (1.495)	**0.015** ^**‡**^
S100	4.286 (2.806)	6.358 (3.567)	**0.004** ^**†**^
LAMP3	0.263 (0.275)	0.269 (0.566)	0.377^‡^
CD83	1.215 (1.323)	1.084 (1.081)	0.709^‡^

pdALN^-^ = Patients without Axillary Lymph Node metastasis at diagnosis. pdALN^+^ = Patients with Axillary Lymph Node metastasis at diagnosis. ALN^-^ = Non-metastatic Axillary Lymph Node.

Data from quantitative features are presented as the mean (standard deviation) or median (interquartile range) and analyzed using Student’s t-test^†^ or the Mann–Whitney U-test^‡^ for normally and non-normally distributed data, respectively. Immune population concentrations are expressed as the percentage of positively stained area within the image (unitless). A *p*-value < 0.05, in bold, indicates statistically significant differences between pdALN^-^ and pdALN^+^.

**Table 3:** Demographic and clinicopathological features of pdALN^-^ and pdALN^+^ from Dataset 2

Variables	pdALN^-^ (*n* = 204)	pdALN^+^ (*n* = 140)	*p*-value
Age (years)	63.00 (16.00)	62.50 (24.00)	0.977^‡^
Tumor diameter (mm)	14.50 (12.00)	21.50 (14.80)	**< 0.001** ^**‡**^
Histological grade			
I	70 (34.31%)	16 (11.43%)	< 0.001*
II	103 (50.49%)	72 (51.43%)	
III	31 (15.20%)	52 (37.14%)	
Perineural invasion			
Positive	30 (14.71%)	52 (37.14%)	< 0.001*
Negative	174 (85.29%)	88 (62.86%)	
ER expression			
Positive	200 (98.04%)	135 (96.43%)	0.494*
Negative	4 (1.96%)	5 (3.57%)	
PR expression			
Positive	177 (86.76%)	120 (85.71%)	0.780*
Negative	27 (13.24%)	20 (14.29%)	
HER2 expression			
Amplified	20 (9.80%)	22 (15.71%)	0.100*
Non-amplified	184 (90.20%)	118 (84.29%)	
Ki67 degree			
Low	104 (50.98%)	52 (37.14%)	0.025*
Medium	41 (20.10%)	30 (21.43%)	
High	59 (28.92%)	58 (41.43%)	
Molecular subtype			
Luminal A	127 (62.25%)	77 (55.00%)	0.178*
Luminal B	77 (37.75%)	63 (45.00%)	

pdALN^-^ = Patients without Axillary Lymph Node metastasis at diagnosis. pdALN^+^ = Patients with Axillary Lymph Node metastasis at diagnosis. ER = Estrogen Receptor. PR = Progesterone Receptor. HER2 = Human Epidermal growth factor Receptor 2. Ki67 = Proliferation index.

Data from quantitative features are presented as the mean (standard deviation) or median (interquartile range) and analyzed using Student’s t-test^†^ or the Mann–Whitney U-test^‡^ for normally and non-normally distributed data, respectively. Data from qualitative features are presented as absolute frequencies (percentages) and analyzed using the chi-square test or, if any expected frequency was less than 5, Fisher’s exact test*. A *p-*value < 0.05, in bold, indicates statistically significant differences between pdALN^-^ and pdALN^+^.

After checking the dataset balance, the features of pdALN^+^ and pdALN^−^ in the two datasets were compared. In Dataset 1, pdALN^+^ had a higher frequency of perineural invasion, a larger tumor diameter, and a higher histological grade than pdALN^−^ (Table 1). Regarding immune cell concentrations, pdALN^+^ cases had higher levels of intratumoral CD21^+^ follicular DCs and LAMP3^+^ DCs, as well as higher levels of CD8^+^ cytotoxic T lymphocytes, and S100^+^ interdigitant DCs in the ALN^−^, but lower levels of CD123^+^ plasmacytoid DCs in ALN^−^, than pdALN^−^ cases (Table 2). Similarly, pdALN^+^ cases of Dataset 2 had a higher frequency of perineural invasion, a larger tumor diameter, a higher histological grade, and a higher Ki67 degree than pdALN^−^ cases (Table 3).

**Fig. 2:**
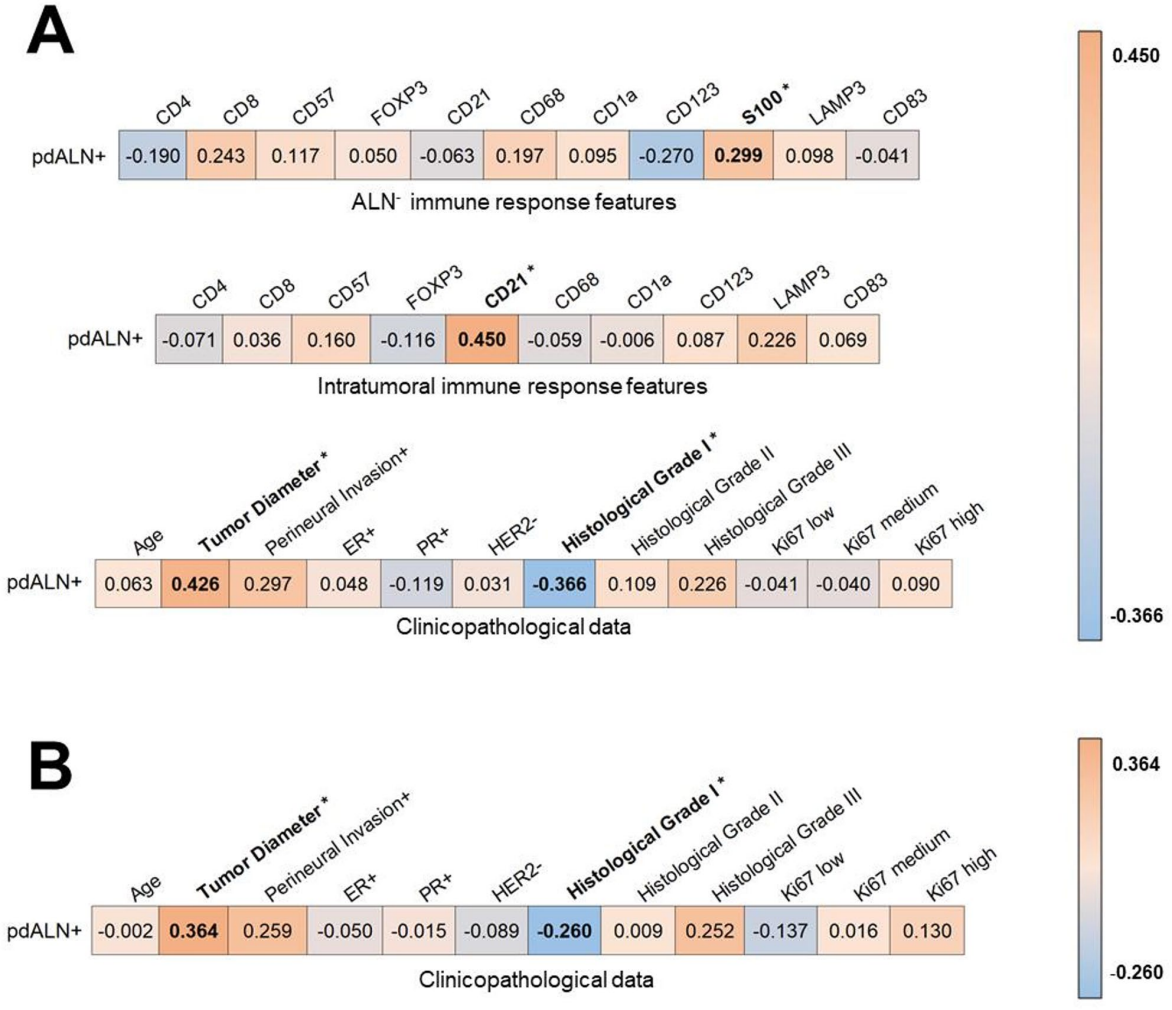
Spearman correlation coefficients of pdALN^+^ and features. Spearman correlation coefficients of pdALN^+^ and ALN^−^ immune response features, intratumoral immune features, and clinicopathological data in **A** Dataset 1. Spearman correlation coefficients of pdALN^+^ and clinicopathological data in **B** Dataset 2. Positive and negative correlations are depicted in orange and blue, respectively; color intensities reflect the strength of the correlations. Features in bold and with * indicate values within the top 10 percent

The correlations between the presence of ALN^+^ at diagnosis and the various features were evaluated (Fig. 2). In Dataset 1, the cutoff based on the top 10 percent corresponded to an absolute Spearman correlation coefficient of 0.298. Accordingly, in Dataset 1, the features most strongly correlated with pdALN^+^ were S100^+^ interdigitant DCs in the ALN^−^, intratumoral CD21^+^ follicular DCs, tumor diameter, and histological grade I (Fig. 2 A). Similarly, in Dataset 2, the cutoff corresponded to an absolute Spearman correlation coefficient of 0.260, identifying tumor diameter and histological grade I as the features most strongly correlated with pdALN^+^ (Fig. 2 B). In both datasets, histological grade I was the only significant feature, being inversely correlated with pdALN^+^.

**Fig. 3:**
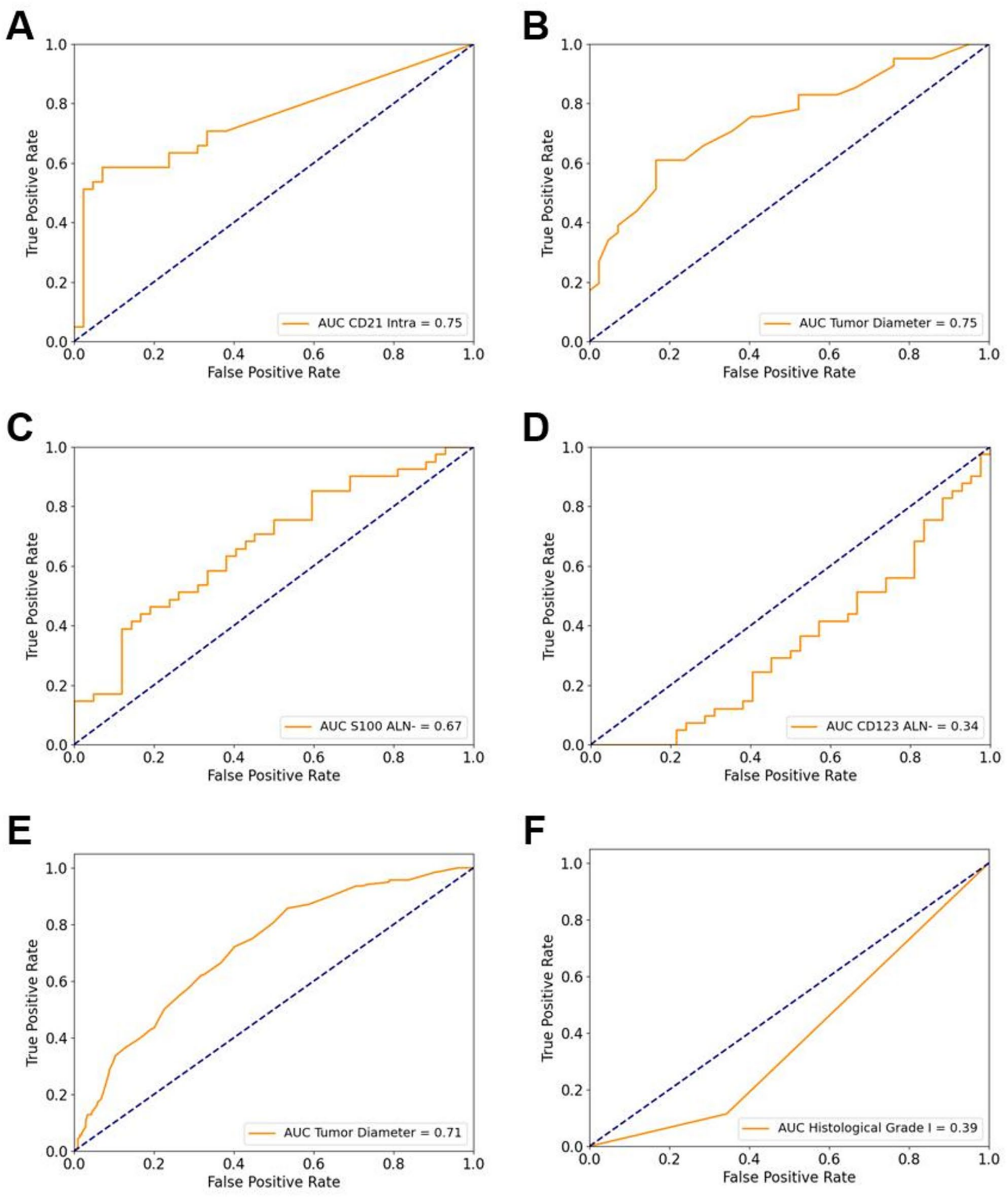
ROC curves based on univariate analysis of the features with an AUC within the top 10 percent for Dataset 1 and Dataset 2. ROC curve of **A** intratumoral CD21^+^ follicular DCs, **B** tumor diameter, **C** S100^+^ interdigitant DCs in the ALN^−^, and **D** CD123^+^ plasmacytoid DCs in the ALN^−^, of Dataset 1, **E** tumor diameter and **F** histological grade I of Dataset 2

In parallel, a univariate ROC curve analysis evaluated the capacity of each feature to predict pdALN^+^. The best features were selected in both datasets using the top 10 percent as a cutoff, with AUC thresholds of 0.655 (Dataset 1) and 0.614 (Dataset 2). Intratumoral CD21^+^ follicular DCs, tumor diameter, S100^+^ interdigitant DCs and CD123^+^ plasmacytoid DCs in the ALN^−^, were the features with the highest individual predictive capacity in Dataset 1 (Fig. 3 A-D); tumor diameter and histological grade I were the features with the best individual predictive capacity in Dataset 2 (Fig. 3 E, F). CD123^+^ plasmacytoid DCs in the ALN^−^ from Dataset 1 and histological grade I from Dataset 2 were the only significant features with an AUC value less than 0.5, indicating an inverse association with ALN^+^ at diagnosis (Fig. 3 D, F).

### Machine learning model performance

The ML models were subsequently developed, including 100 repetitions of the computational procedure with random states. These generated a large quantity of results, so only two significant examples, Example 1 (random state 0) and Example 2 (random state 42), are presented here to illustrate the outcomes. Model 2 was eventually chosen for its robustness.

**Fig. 4:**
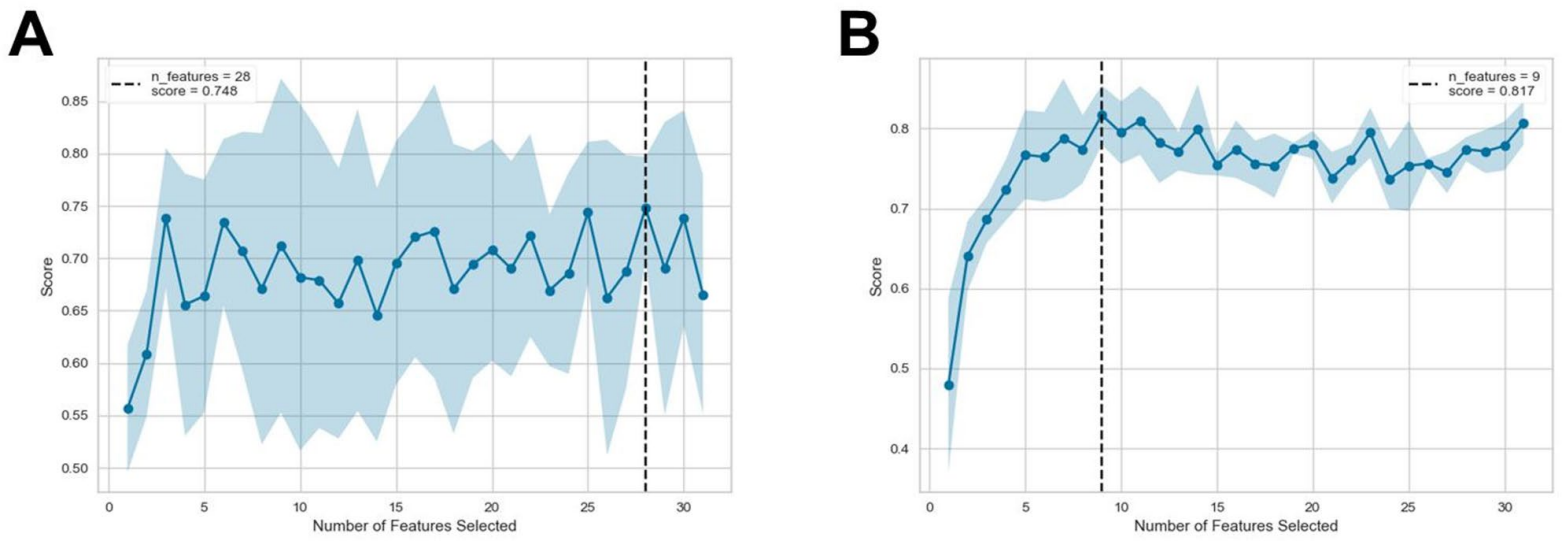
Representative examples of feature selection in Model 2. The shaded area indicates cross-validation variability, and each blue dot to the left of the dotted line represents a feature included in the model. The scoring metric is AUC. Example 1 **A** corresponds to random state 0, and Example 2 **B** corresponds to random state 42

As with the feature selection, the random states produced highly variable outcomes. While Example 1 (Fig. 4 A) yielded 28 optimal features, with an AUC value of 0.748, Example 2 (Fig. 4 B) produced nine optimal features, with a higher AUC value of 0.817.

**Fig. 5:**
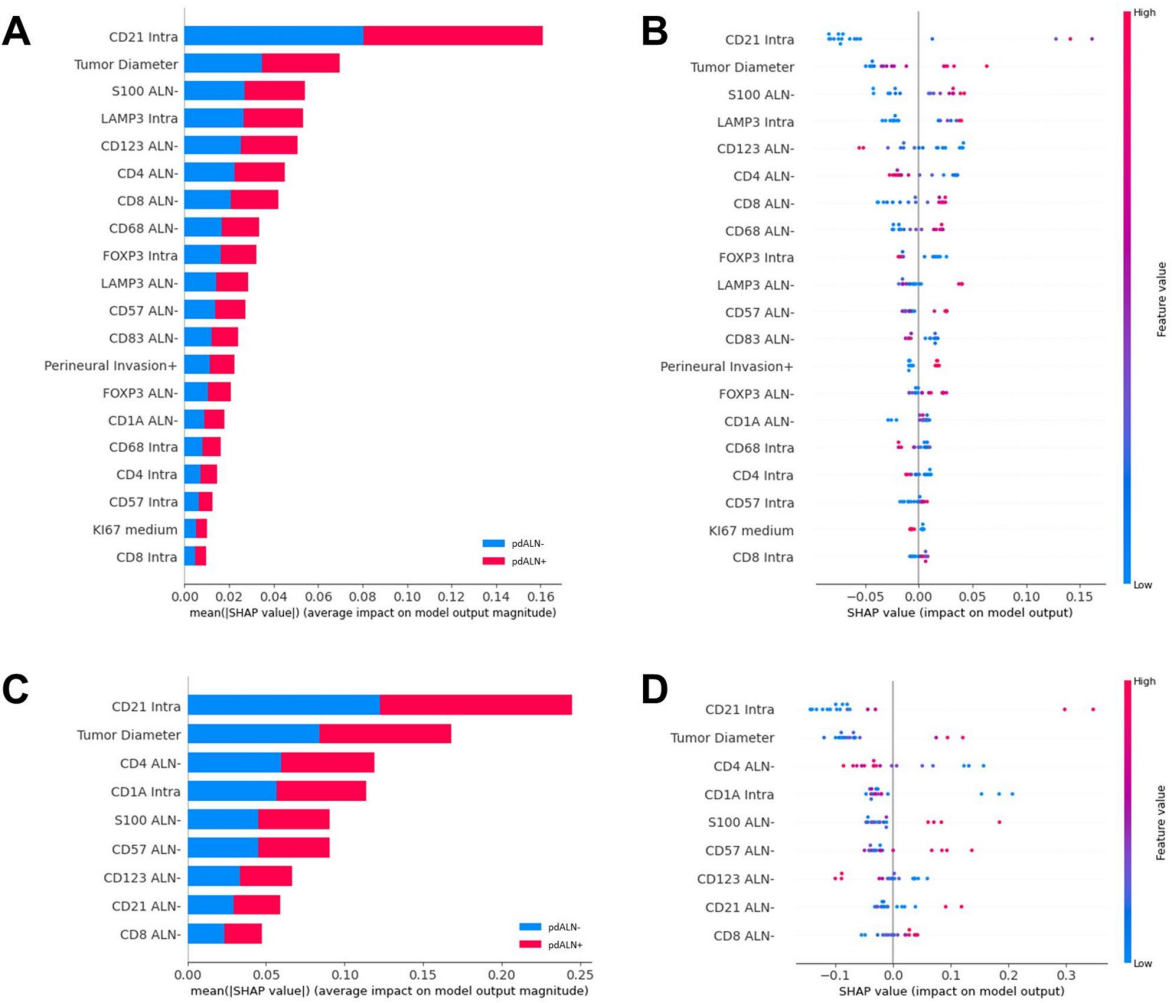
Representative examples of Model 2 for the result explainability employing SHAP values. Example 1 **A** corresponds to random state 0, and Example 2 **C** corresponds to random state 42. The SHAP bar plots **A** and **C** display the mean absolute SHAP values for each feature. Each bar represents a feature, and the length of the bar indicates the average absolute contribution of that feature to the model decision-making process. The SHAP summary plot (beeswarm plot) combines feature importance with the direction and distribution of impacts for individual predictions **B**, **D**. The position of each point along the x-axis indicates the impact of that feature on the prediction for a particular patient. A wider spread signifies varying importance levels of that feature across the dataset. Each point represents a patient, and the color of the points indicates the value of the feature for that patient (blue for low values, red for high values). Features with points shifted to the left side of the plot (negative SHAP values) suggest that the feature reduces the risk of ALN^+^ presence at diagnosis, while those shifted to the right (positive SHAP values) suggest that the feature increases the predicted risk. Example 1 **B** corresponds to random state 0, and Example 2 **D** corresponds to random state 42

With respect to explainability, the feature with the biggest weight in decision-making in both examples was intratumoral CD21^+^ follicular DCs, followed by the tumor diameter (Fig. 5 A, C). Remarkably, these two features were consistently the most influential in the classification, across nearly all random states.

The next most influential features were S100^+^ interdigitant DCs in the ALN^−^ and intratumoral LAMP3^+^ DCs, in Example 1 (Fig. 5 A). In Example 2, the next most important features were CD4^+^ helper T lymphocytes in the ALN^−^ and intratumoral CD1a^+^ Langerhans DCs (Fig. 5 C). In general, the features with higher concentrations corresponding to positive SHAP values in the beeswarm plots (Fig. 5 B, D) corresponded to those with positive Spearman correlation coefficients and AUC values greater than 0.5, as with the discrimination of pdALN^+^. Examples of such features include intratumoral CD21^+^ follicular DCs and tumor diameter. Conversely, the features with lower concentrations corresponding to positive SHAP values in the beeswarm plots (Fig. 5 B, D) had a negative Spearman correlation coefficient and an AUC value less than 0.5, as with the discrimination of pdALN^+^. Instances of such features include CD4^+^ helper T lymphocytes and CD123^+^ plasmacytoid DCs in the ALN^−^.

**Table 4:** Performance of Models 1, 2, and 3 as measured by the median values of AUC, accuracy, and recall values after 100 repetitions

	AUC	Accuracy	Recall
Model 1	0.63 (0.19)	0.65 (0.18)	**0.62 (0.25)**
Model 2	**0.79 (0.14)**	**0.71 (0.12)**	**0.62 (0.25)**
Model 3	0.69 (0.07)	0.64 (0.06)	0.50 (0.11)
*p*-value	< 0.001**	< 0.001**	< 0.001**

AUC = Area Under the Curve. Model 1 = Model integrating exclusively clinicopathological features from Dataset 1. Model 2 = Model integrating clinicopathological features and immune population from Dataset 1. Model 3 = Model integrating clinicopathological features from Dataset 2.

Data are presented as the median (interquartile range) and compared using Friedman’s test**. Best-performing values per metric, in bold, are highlighted based on the highest median. A *p*-value < 0.05, in bold, indicates statistically significant differences between models for each metric.

**Table 5:** Pairwise comparisons of Models 1, 2, and 3 with respect to the median values of AUC, accuracy, and recall

Metric	Comparison	*p*-value	
AUC	Model 1 vs. **Model 2**	**< 0.001** ^**††**^	
	Model 1 vs. **Model 3**	**0.012** ^**††**^	
	**Model 2** vs. Model 3	**< 0.001** ^**††**^	
Accuracy	Model 1 vs. **Model 2**	**< 0.001** ^**††**^	
	Model 1 vs. Model 3	0.630^††^	
	**Model 2** vs. Model 3	**< 0.001** ^**††**^	
Recall	Model 1 vs. Model 2	**< 0.001** ^**††**^	
	**Model 1** vs. Model 3	**0.002** ^**††**^	
	**Model 2** vs. Model 3	**< 0.001** ^**††**^	

AUC = Area Under the Curve. Model 1 = Model integrating exclusively clinicopathological features from Dataset 1. Model 2 = Model integrating clinicopathological features and immune population from Dataset 1. Model 3 = Model integrating clinicopathological features from Dataset 2.

Comparisons are made using Conover’s test^††^. Entries in bold indicate the best-performing model in each comparison, highlighted only when the differences are statistically significant. A *p*-value < 0.05, in bold, indicates statistically significant differences between model pairs for each metric.

To evaluate the models further and identify the best performer, the median recall, accuracy, and AUC values were compared across the three models using Friedman’s test. The results indicated significant differences for all metrics. Model 2, which included the immune response features and clinicopathological data, outperformed Models 1 and 3 (Table 4). Subsequent comparisons using Conover’s test pinpointed significant pairwise differences in all estimates of AUC, accuracy, and recall between the models, except for those of the accuracy of Models 1 and 3 (Table 5).

**Fig. 6:**
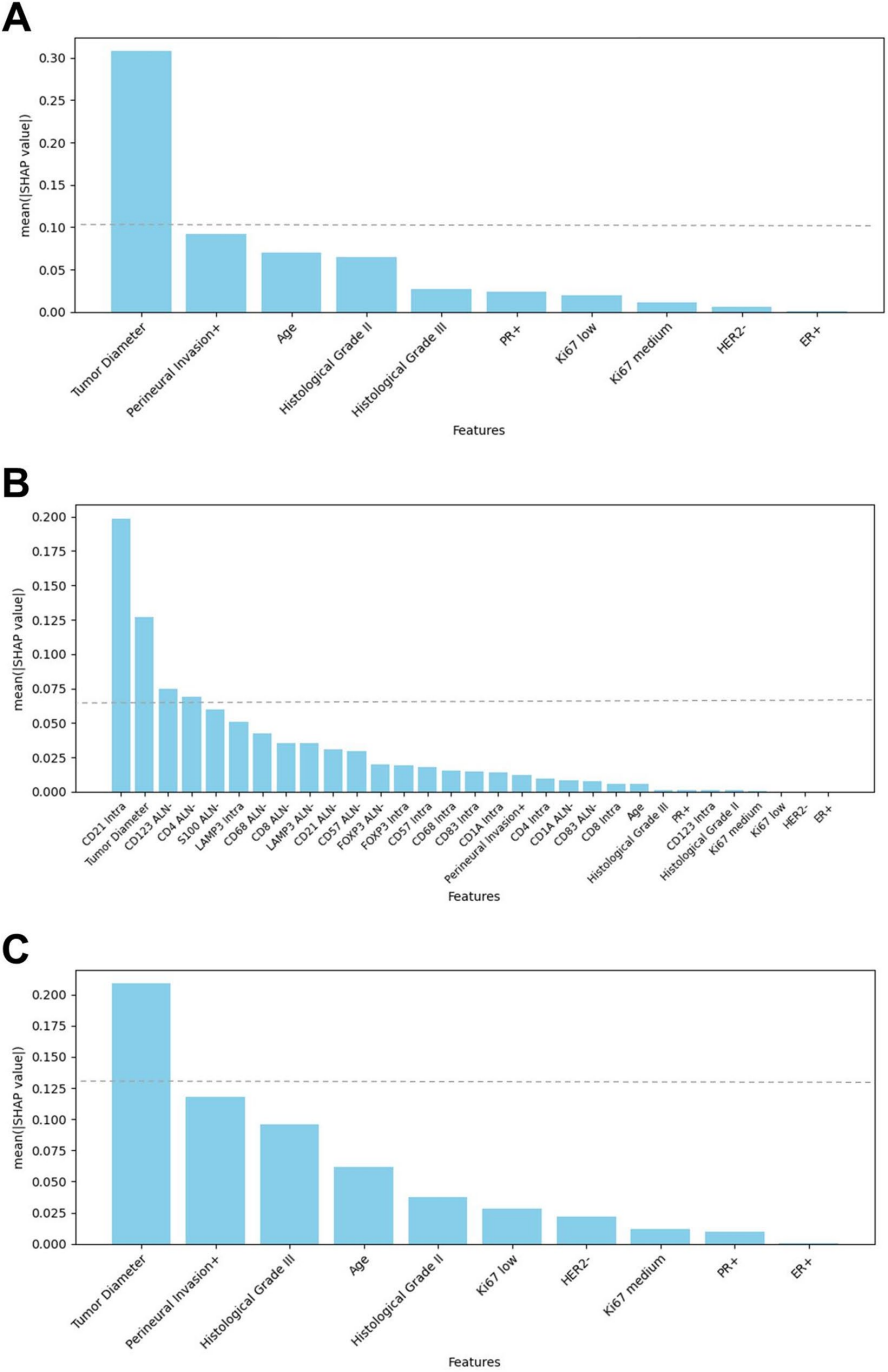
Mean SHAP values of the 100 repetitions for each feature. **A** Model 1, **B** Model 2, and **C** Model 3. The features with SHAP values above the dashed lines fall within the top 10 percent

Based on the top 10 percent of SHAP values, the feature with the most significant influence in the model’s decision-making process was tumor diameter in Models 1 and 3 (Fig. 6 A, C). Intratumoral CD21^+^ follicular DCs, tumor diameter, CD123^+^ plasmacytoid DCs and CD4^+^ helper T lymphocytes in the ALN^−^ were the features that contributed most strongly to the decision-making process for Model 2 (Fig. 6 B).

#### Final machine learning model

**Fig. 7:**
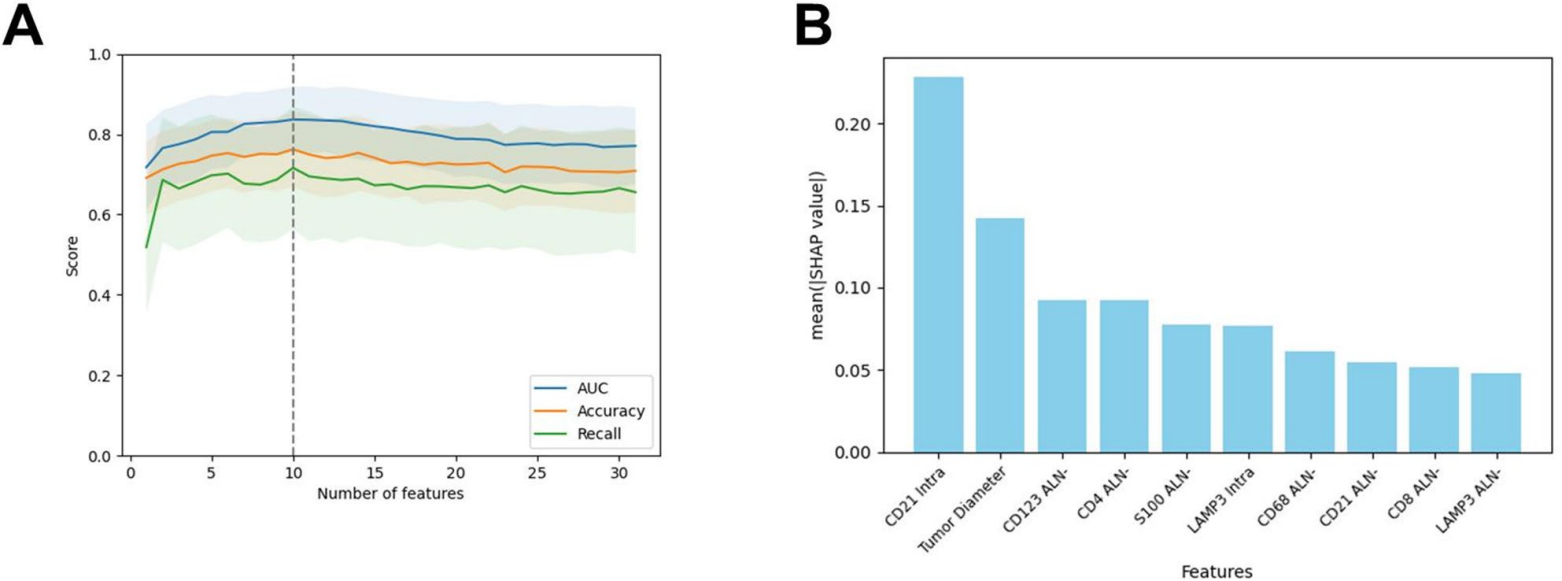
Model 4 metrics and mean SHAP values. **A** AUC (blue line and shaded area), accuracy (orange line and shaded area), and recall (green line and shaded area) values depending on the number of features included in Model 4. Solid lines represent the mean values from the 100 repetitions, while shaded areas of the same color indicate the standard deviation. The dashed line highlights the optimal number of features. **B** Mean SHAP values of the 100 repetitions for each feature included in Model 4

After identifying the best-performing model (Model 2), Model 4 was developed, for which an optimal cutoff was determined for ten features using mean and median (Fig. 7 A, only mean values shown). Of these ten features, intratumoral CD21^+^ follicular DCs, and tumor diameter had the highest SHAP values (Fig. 7 B). Incorporating these into Model 4 produced a median AUC of 0.84, a median accuracy of 0.76, and a median recall of 0.75, all three values thereby outperforming those of Model 2 (Table 6).

**Table 6:** Performance of Models 2 and 4 with respect to the median AUC, accuracy, and recall values after 100 repetitions

	AUC	Accuracy	Recall
Model 2	0.79 (0.14)	0.71 (0.12)	0.62 (0.25)
Model 4	**0.84 (0.11)**	**0.76 (0.12)**	**0.75 (0.26)**
*p*-value	< 0.001**	< 0.001**	< 0.001**

AUC = Area Under the Curve. Model 2 = Model integrating clinicopathological features and immune population from Dataset 1. Model 4 = Model with the optimal ten features, integrating clinicopathological features and immune population from Dataset 1.

Data are presented as the median (interquartile range) using the Wilcoxon signed-rank test**. Best-performing values per metric, in bold, are highlighted based on the highest median. A *p*-value < 0.05, in bold, indicates statistically significant differences between models for each metric.

## Discussion

BC prognosis is currently based on well-established clinicopathological factors, including tumor size, histological grade, molecular subtype, lymph node involvement, and metastatic state [50]. This BC staging system, a worldwide gold standard among pathologists [26], aids patient stratification at diagnosis, providing clinical guidelines for treatment personalization, which can ultimately improve patient outcomes.

In the search for predictive biomarkers, increasing attention has been paid to the elements within the tumor microenvironment, particularly immune cells, which have been shown to significantly influence cancer progression and patient prognosis [51]. However, the primary tumor microenvironment might not entirely reflect the metastatic status. Therefore, understanding the spread of the tumor cells to lymph nodes and, eventually, to distant organs, also requires an examination of the ALN^−^ and ALN^+^ microenvironments. Indeed, lymph nodes are often the first site of BC metastasis, and the crosstalk between tumor cells and immune cells in the primary tumor and ALNs plays a key role in this metastatic process [10]. Thus, identifying reliable immune biomarkers is crucial to improving patient stratification and developing more precise treatments [6, 52, 53]. For this purpose, four ML models were developed using clinicopathological features and immune populations from the primary tumor and ALN^−^ to identify the factors most closely associated with pdALN^+^.

This is the first study to combine clinicopathological and immune features to predict ALN^+^ at diagnosis in BC patients using ML models. These features were previously evaluated by our group using established statistical models [11–13]. Unlike those, ML models offer the advantages of capturing non-linear relationships and complex patterns [14] and generalizing learned patterns to new cases without retraining.

A key aspect of ML implementation is the preprocessing phase, with a check of data balance being one of the main steps. In this case, Dataset 1 was roughly balanced, with an equal number of pdALN^+^ and pdALN^−^ cases, while Dataset 2 showed a slight imbalance (40.7% of pdALN^+^). A balanced dataset is important for mitigating potential bias towards the majority class during training [54]. Therefore, the Synthetic Minority Oversampling Technique (SMOTE) was applied to assess the impact of data imbalance on the performance of Model 3 [55]. The entire computational procedure was conducted with and without SMOTE for Model 3. The results were slightly worse with SMOTE, probably because of the smaller quantity of real data, and the error introduced by synthetic data. Consequently, SMOTE was not used in the final analysis of Model 3 since the imbalance was not great enough to require a balancing technique. Other essential steps in the preprocessing phase included descriptive, Spearman correlation, and ROC curve analyses. Some features of the two datasets were significantly correlated with pdALN^+^ and showed adequate predictive capacity, providing sufficient explanatory power to proceed with further modeling.

Selecting a suitable ML algorithm improves prediction metrics. Thus, several ML algorithms were evaluated, including Decision Tree, RF, and eXtreme Gradient Boosting. RF performed slightly better than the others (Supplementary material Table S2). Accordingly, the three ML models were subsequently developed using the RF classifier.

The differences in performance between the three models can be attributed to variations in dataset size and features. Model 3 was trained on a dataset approximately four times that of the other two models, which led to greater stability and a lower interquartile range across all metrics. In contrast, Model 1 and Model 2 were more sensitive to test-set variability. Nevertheless, adding immune population data compensates for the smaller dataset size, so Model 2 outperformed Model 1 and Model 3 in terms of accuracy and AUC. Along with those improvements, integrating immune data into Model 2 shifted tumor diameter from the first- to the second-ranking position (although it remained within the top 10 percent) and other clinicopathological features moved to the lower positions. These results highlight the crucial role of the immune response in predicting pdALN^+^, as observed in other studies, where incorporating additional data types (such as radiomics data) improved model performance [56]. Despite their improved overall classification performance, Models 1 and 2 achieved similar recall, suggesting that, in this case, the immune data enhance discrimination by reducing false positives rather than by improving the detection of true positives. Moreover, Model 1 achieved better recall than Model 3, probably because the latter proved to be more conservative, prioritizing precision over recall. The two models had similar accuracy, suggesting that the performance might have reached a plateau with the available clinicopathological data, making tumor diameter the most important feature for prediction in both models, regardless of dataset size.

Having determined that Model 2 performed best, Model 4 was developed using the same dataset and features. Similar to Models 1, 2 and 3, ten features were recognized as the optimal number, and the 100 repetitions with different random states were conducted using this feature set. This approach reduced variability and improved performance, resulting in greater stability and a narrower interquartile range for all metrics compared with Model 2. Consequently, Model 4 outperformed Model 2, demonstrating its potential as an ML-based stratification tool in BC and its enhanced ability to detect true positives.

In line with our Model 3, Jia-Long Wu et al. [24] developed an ML-based model using the Support Vector Machine classifier and clinicopathological data of the primary tumor to predict ALN involvement in 1325 BC patients. While their model performed slightly better than our Model 3, it underperformed compared with our Model 4 in terms of its accuracy (0.74 vs. 0.64 vs. 0.76) and AUC (0.77 vs. 0.69 vs. 0.84). Interestingly, their model performed slightly better with respect to recall (0.76 vs. 0.50 vs. 0.75). The overall improved performance of Model 4 may be attributed to the inclusion of immune response-related features, which probably provided additional predictive power. Moreover, we hypothesize that the larger sample size and the inclusion of the lymphovascular invasion feature may account for the better outcome of the Jia-Long Wu et al. [24] model compared with that of Model 3. Besides tumor diameter, lymphovascular invasion is a factor known to be associated with BC prognosis, although it was excluded from the proposed models because its prevalence may be overestimated by pathologists [57]. Except in the case of lymphovascular invasion, tumor size and histology grade were consistently found to be important factors in predicting pdALN^+^. These factors have been reported to be predictive of pdALN^+^ in earlier studies [58, 59]. In this context, Vrdojlak et al. [60] developed an ML-based model to predict pdALN^+^ using clinicopathological records in a dataset of 8381 BC patients, with RF emerging as one of the best-performing algorithms. The AUC was slightly higher than that of Model 3 but lower than that of Model 4 (0.76 vs. 0.69 vs. 0.84). The difference in AUC values between their model and our Model 3 may be attributed to variations in the distribution of histological grades, given that 65% of the patients in their dataset had grade III tumors, compared with only 24% in our proposed models. Furthermore, the two datasets used in our study included only Luminal patients, while their study covered all molecular subtypes of BC. In addition, the improved performance of Model 4 was probably due to the introduction of immune response features, which appear to add significant value to BC stratification. Similar to Model 3, tumor size was the most important predictor in the model developed by Vrdojlak et al. [60], but they also identified Ki67 and patient age as predictors. Tumor diameter was also ranked as the second-most important predictor in Model 4. It is of note that, as previously mentioned, Model 4 achieved a slightly better AUC than the models developed by Wu et al. [24] and Vrdojlak et al. [60], highlighting the importance of the immune response in predicting pdALN^+^. Other studies have developed ML models to predict ALN^+^ at diagnosis in BC patients using genetic [61, 62] and radiomic features [56, 63–65], but none of them used immune data.

Of the ten features identified in the current study, nine were related to the immune response, further reinforcing the importance of these populations. Seven of the nine immune-related features were found in the ALN^−^, and six were DC subsets. Despite their relevance in this work, the immune content in ALN^−^ and the predictive role of DCs in pdALN^+^ remain underexplored. Moreover, the role of many immune markers in pdALN^+^ is not well-documented, but our group’s research has provided valuable insights in this area [11–13]. Although CD123^+^ in the ALN^−^ has not been significantly associated with pdALN^+^ [66], our earlier work suggested that CD123^+^ in the ALN^−^ acts as a protective factor against pdALN^+^ [11], which is consistent with the findings of the univariate ROC curves, the correlation study, the descriptive analysis, and the beeswarm plots of the current study. Similarly, S100^+^ in the ALN^−^ has been very rarely documented, but identified as a prognostic marker in this and the previous study [11]. Regarding macrophages, CD68^+^ in the ALN^−^ has been reported as being a poor prognostic factor and a risk factor for pdALN^+^ [11], which is consistent with the findings of the current study. Other immune markers, such as CD21^+^, CD4^+^, CD8^+^, and LAMP3^+^ (CD208) in the ALN^−^, as well as intratumoral LAMP3^+^, have received limited attention in BC research and did not show any significant associations with pdALN^+^ in our earlier study [11]. Although no studies found an association between CD21^+^ and pdALN^+^, CD21^+^ in the primary tumor was identified as a risk factor for pdALN^+^ [11]. Notably, in the present ML-based Model 4, intratumoral CD21^+^ contributed more strongly to the algorithm decision-making than did tumor diameter, making it a promising biomarker candidate. Additionally, as shown in the beeswarm plots, patients with higher concentrations of intratumoral CD21^+^ follicular DCs and a larger tumor diameter were more likely to develop ALN^+^. These results underscore the importance to BC stratification of considering mechanistic features, such as mediating inflammation, not only in the tumor but also in the ALN^−^, in addition to clinicopathological data.

To provide a biological context for these findings, CD21^+^ follicular DCs are not only structural components of germinal centers but have also demonstrated prognostic relevance in several pathological contexts. In follicular lymphoma, the organization and composition of follicular DC networks are key elements of the TME that influence clinical outcome [67]. Similarly, expansion of CD21^+^ follicular DCs meshworks represents a defining microenvironmental feature in angioimmunoblastic T-cell lymphoma [68]. Beyond hematologic malignancies, the presence of mature tertiary lymphoid structures characterized by CD21^+^ follicular DCs has emerged as an immune biomarker across multiple solid tumors [69]. These observations support the biological plausibility that intratumoral CD2^+^ follicular DCs identified in our cohort may reflect functionally organized immune niches with clinical relevance. The organization of these structures may be guided by chemokines such as CXCL13, which is expressed by follicular DCs and attracts both B cells and T follicular helper cells to form functional lymphoid aggregates [70]. While CXCL13 is critical for the initiation of tertiary lymphoid structures, its role in their maintenance appears to be less absolute [70], suggesting that once established, CD21^+^ networks could persist and modulate local immune responses independently. This raises the possibility that the intratumoral CD21^+^ cells observed in our cohort reflect similar chemokine-driven lymphoid organization, potentially contributing to lymphatic dissemination.

From a translational perspective, immune markers such as CD21^+^ follicular DCs can be assessed by IHC, making their implementation in routine pathology technically feasible and achievable at relatively low cost per sample. Nevertheless, variability related to staining protocols, antibody selection, and inter-observer interpretation may affect reproducibility. The integration of digital pathology and automated image analysis could solve these limitations by enabling standardized and quantitative assessment, thereby facilitating clinical adoption. However, additional wet-lab studies will be required to support clinical translation.

Despite achieving significant results, some limitations that could affect the interpretation and generalization of the results must be acknowledged. First, the study was restricted to Luminal BC subtypes due to the underrepresentation and high heterogeneity of triple-negative and HER2-enriched tumors. Consequently, the developed models may not generalize to non-Luminal subtypes. Furthermore, approximately 10.8% of patients underwent neoadjuvant treatment, a known modulator of tumor and nodal immune microenvironments that may introduce confounding signaling patterns. Additionally, the lack of data on comorbidities, which significantly influence disease progression and contribute to pdALN^+^ [71, 72], may have limited the model’s ability to predict pdALN^+^. Moreover, the small size of our datasets, as mentioned earlier, increased results variability and limited the statistical power of our analyses, potentially affecting model’s robustness.

Another important limitation is the lack of independent external validation. Although several external datasets similar to Datasets 1 and 2 have been reported (Supplementary Material Table S3), to our knowledge, no publicly available, independent BC datasets include the full range of features analyzed in the present study. An exploratory evaluation using the two most similar external datasets available was conducted. However, differences in available features and cohort characteristics limited the interpretability of these results, further underscoring the critical need for large, comprehensive, publicly available BC datasets. Consequently, Models 1–4 were evaluated using internal validation only. While appropriate for preliminary assessment, internal validation may capture cohort-specific clinical, biological, or technical characteristics that influence model parameters and performance estimates. As a result, extrapolation of these findings to independent patient populations should be approached with caution. Future validation in large, independent, multicenter cohorts will be essential to confirm the stability and generalizability of these models.

Beyond the need for external validation, several methodological refinements could further strengthen the robustness and applicability of the proposed models. In particular, excluding patients who received neoadjuvant treatment and incorporating comorbidity information would help reduce potential confounding effects. Integrating complementary radiological and genetic data may also enhance predictive performance. Moreover, increasing the sample size and expanding the representation of triple-negative and HER2-enriched molecular subtypes may enable the development of models that include all BC subtypes, thereby reducing subtype-related bias and improving broader applicability. However, assembling large, comprehensive medical datasets remains challenging, especially for these less common molecular subtypes. Nevertheless, the novel biomarker candidates identified herein open up new avenues of mechanistic modeling, enabling exploration of the biological mechanisms underlying the immune response not only in the primary tumor, but also in ALN^−^ samples of BC patients.

Since prior studies demonstrated differences in immune concentrations between matched ALNs (ALN^+^ vs. ALN^−^) in pdALN^+^ in the Luminal A and triple-negative BC subtypes [73], future investigations could expand on the experimental framework established in the present study. This could be achieved by including ALN^+^ nodes and comparing them with ALN^−^ nodes within the pdALN^+^ subgroup, providing further insights into ALN immune dynamics and their potential clinical implications.

## Conclusions

This study demonstrates the power of integrating immune response into ML models for predicting pdALN^+^, achieving a median AUC of 0.84, a median accuracy of 0.76, and a median recall of 0.75. Among the identified features, intratumoral CD21^+^ follicular dendritic cells emerged as the most predictive marker, outperforming well-established clinicopathological prognostic factors such as tumor diameter. The strong predictive value of intratumoral CD21^+^ suggests its critical role in modulating the tumor microenvironment and influencing metastatic progression. These findings position intratumoral CD21^+^ not only as a promising prognostic biomarker but also as a potential candidate for pdALN^+^ risk stratification, early detection, and personalized therapeutic strategies. Future clinical studies should validate its potential as a therapeutic target and assess its utility in guiding clinical decision-making.

## Supplementary Information

Below is the link to the electronic supplementary material.

Supplementary Material 1

## Data Availability

The data supporting the findings of this study are not publicly available due to the potential risk of compromising the privacy of research participants; however, they can be obtained from the corresponding author (CLP) upon reasonable request. The code developed for this study is publicly available. The latest version can be accessed on GitHub: https://github.com/bmmbUPF/breast-cancer-alnm-prediction.

## Abbreviations

AI: Artificial intelligence
AUC: Area under the curve
ALN: Axillary lymph node
ALN^+^: Axillary lymph node with metastasis
ALN^−^: Axillary lymph node without metastasis
BC: Breast cancer
CV: Cross-validation
pdALN^+^: Patients with ALN^+^ at diagnosis
pdALN^−^: Patients with ALN^−^ at diagnosis
DC: Dendritic cell
ER: Estrogen receptor
HER2: Human epidermal growth factor receptor 2
HUTVC: Hospital universitari de tortosa verge de la cinta
HJ23: Hospital universitari joan XXIII
IHC: Immunohistochemical
Ki67: Proliferation index
ML: Machine learning
PR: Progesterone receptor
RF: Random forest
RFE: Recursive feature elimination
ROC: Receiver operating characteristic
SMOTE: Synthetic minority oversampling technique
SHAP: Shapley additive explanations
