# Supplementary material for: Leveraging immune and clinicopathological profiles with machine learning to predict axillary lymph node metastasis in breast cancer patients
Source: Breast Cancer Res. 2026 May 8;28:121. doi: 10.1186/s13058-026-02294-9 (PMC13321410; doi:10.1186/s13058-026-02294-9)
Supplement: Supplementary file 1 — Supplementary Material 1 [file 13058_2026_2294_MOESM1_ESM.pdf]

Supplementary Material – ***Leveraging immune and clinicopathological profiles with machine learning to predict axillary lymph node metastasis in breast cancer patients***

1. Feature selection method

Table S1. Performance of different feature selection methods across the three classification models as measured by the median values of AUC, accuracy, and recall values after 100 repetitions.

		AUC	Accuracy	Recall
**Model 1**	SFS-AUC	0.611 (0.174)	0.588 (0.118)	0.500 (0.120)
	SFS-accuracy	0.601 (0.184)	0.588 (0.176)	0.500 (0.240)
	VIF	**0.667 (0.167)**	**0.647 (0.176)**	0.620 (0.250)
	L1-LR	0.639 (0.168)	0.588 (0.176)	0.620 (0.250)
	RFECV-AUC	0.632 (0.191)	**0.647 (0.176)**	0.620 (0.250)
	RFECV-accuracy	0.646 (0.149)	**0.647 (0.176)**	**0.620 (0.220)**
**Model 2**	SFS-AUC	0.750 (0.153)	0.706 (0.176)	0.620 (0.250)
	SFS-accuracy	0.736 (0.167)	0.676 (0.118)	0.620 (0.250)
	VIF	0.778 (0.139)	0.706 (0.176)	0.620 (0.250)
	L1-LR	0.778 (0.149)	0.706 (0.176)	0.620 (0.250)
	RFECV-AUC	**0.792 (0.139)**	**0.706 (0.118)**	0.620 (0.250)
	RFECV-accuracy	0.778 (0.167)	**0.706 (0.118)**	**0.620 (0.130)**
**Model 3**	SFS-AUC	0.665 (0.068)	**0.638 (0.058)**	0.460 (0.150)
	SFS-accuracy	0.655 (0.087)	**0.638 (0.058)**	0.430 (0.140)
	VIF	0.694 (0.072)	0.638 (0.072)	0.500 (0.130)
	L1-LR	**0.699 (0.078)**	**0.638 (0.058)**	0.500 (0.170)
	RFECV-AUC	0.691 (0.072)	**0.638 (0.058)**	**0.500 (0.110)**
	RFECV-accuracy	0.681 (0.087)	0.623 (0.083)	0.500 (0.140)

Data are presented as the median (interquartile range). Best-performing values per metric, in bold, are highlighted based on the highest median and the lowest interquartile range.

Abbreviations: AUC = Area Under the Curve. Model 1 = Model integrating exclusively clinicopathological features from Dataset 1. Model 2 = Model integrating clinicopathological features and immune population from Dataset 1. Model 3 = Model integrating clinicopathological features from Dataset 2. SFS-AUC = Sequential Feature Selector using Area Under the Curve scoring. SFS-accuracy = Sequential Feature Selector using accuracy scoring. VIF = Variance Inflation Factor. L1-LR = L1-Regularized Logistic Regression. REFCV-AUC = Recursive Feature Elimination with Cross-Validation using Area Under the Curve scoring. REFCV-accuracy = Recursive Feature Elimination with Cross-Validation using accuracy scoring.

2. Machine learning algorithm

Table S2. Performance of different ML algorithms across the three classification models as measured by the median values of AUC, accuracy, and recall values after 100 repetitions.

	ML algorithm	AUC	Accuracy	Recall
	RF	**0.632 (0.191)**	**0.647 (0.176)**	**0.620 (0.250)**
**Model 1**	XGBOOST	0.625 (0.186)	0.529 (0.118)	0.750 (0.500)
	DT	0.594 (0.172)	0.588 (0.176)	0.500 (0.240)
	RF	**0.792 (0.139)**	**0.706 (0.118)**	0.620 (0.250)
**Model 2**	XGBOOST	0.722 (0.153)	0.647 (0.118)	**0.750 (0.260)**
	DT	0.646 (0.194)	0.647 (0.176)	0.620 (0.250)
	RF	**0.691 (0.072)**	**0.638 (0.058)**	**0.500 (0.110)**
**Model 3**	XGBOOST	0.667 (0.079)	0.587 (0.203)	0.750 (0.610)
	DT	0.589 (0.086)	0.594 (0.058)	0.430 (0.170)

Data are presented as the median (interquartile range). Best-performing values per metric, in bold, are highlighted based on the highest median and the lowest interquartile range.

Abbreviations: ML = Machine Learning. AUC = Area Under the Curve. Model 1 = Model integrating exclusively clinicopathological features from Dataset 1. Model 2 = Model integrating clinicopathological features and immune population from Dataset 1. Model 3 = Model integrating clinicopathological features from Dataset 2. RF = Random Forest. XGBOOST = eXtreme Gradient Boosting. DT = Decision Tree.

3. External datasets

Table S3. Publicly available breast cancer datasets considered for external validation.

	Tumor Diameter	Age	Histological Grade	Perineural Invasion	ER	PR	HER2	Ki67	Nodal Status	Molecular Subtype	Immune Populations	URL
**cBioPortal: METABRIC**	x	x	x		x	x	x		x	x		https://www.cbioportal.org/study/summary?id=brca_metabric
cBioPortal: TCGA-PanCancer Atlas		x							x	x		https://www.cbioportal.org/study/summary?id=brca_tcga_pan_can_atlas_2018
cBioPortal: MSKCC, Clinical Cancer Res 2020	x		x		x		x					https://www.cbioportal.org/study/summary?id=brca_pareja_msk_2020
cBioPortal: SMC 2018		x								x		https://www.cbioportal.org/study/summary?id=brca_smc_2018
cBioPortal: British Columbia, Nature 2012	x	x	x		x	x	x		x			https://www.cbioportal.org/study/summary?id=brca_bccrc
cBioPortal: Broad, Nature 2012		x	x		x	x	x					https://www.cbioportal.org/study/summary?id=brca_broad
cBioPortal: Sanger, Nature 2012		x	x		x	x	x					https://www.cbioportal.org/study/summary?id=brca_sanger

cBioPortal: TCGA, Cell 2015		x		x	x	x		x		https://www.cbioportal.org/study/summary?id=brca_tcga_pub2015
cBioPortal: TCGA, Firehose Legacy		x		x	x	x		x		https://www.cbioportal.org/study/summary?id=brca_tcga
cBioPortal: TCGA, Nature 2012		x		x	x	x		x		https://www.cbioportal.org/study/summary?id=brca_tcga_pub
TCIA: TCGA-Breast-Radiogenomics		x		x	x	x		x		https://www.cancerimagingarchive.net/analysis-result/tcga-breast-radiogenomics/
**Grand Challenge: Early Breast Cancer Core-Needle Biopsy WSI Dataset**	x	x	x	x	x	x	x	x	x	https://bcnb.grand-challenge.org/
Zenodo: SEER Breast Cancer Dataset	x	x	x	x	x			x		https://zenodo.org/records/5120960

Available features are indicated with an “x”, and the two external datasets most similar to the internal datasets are highlighted in bold. Other public data repositories (e.g., Kaggle) and datasets published in Scientific Data were screened to identify potentially suitable external validation cohorts. However, no datasets matching the required features were found.

Abbreviations: ER = Estrogen Receptor. PR = Progesterone Receptor. HER2 = Human Epidermal growth factor Receptor 2. Ki67 = Proliferation index. TCGA = The Cancer Genome Atlas. METABRIC = Molecular Taxonomy of Breast Cancer International Consortium. MSKCC = Memorial Sloan Kettering Cancer Center. SMC = Samsung Medical Center. TCIA = The Cancer Imaging Archive. SEER = Surveillance, Epidemiology, and End Results Program. WSI = Whole Slide Imaging.
